# Presence of lung cancer and high gender, age, and physiology score as predictors of acute exacerbation in combined pulmonary fibrosis and emphysema: A retrospective study

**DOI:** 10.1097/MD.0000000000011683

**Published:** 2018-08-03

**Authors:** Jee Youn Oh, Young Seok Lee, Kyung Hoon Min, Gyu Young Hur, Sung Yong Lee, Kyung Ho Kang, Jae Jeong Shim

**Affiliations:** Division of Pulmonary, Allergy, and Critical Care Medicine, Department of Internal Medicine, Korea University Guro Hospital, Korea University College of Medicine, Seoul, Republic of Korea.

**Keywords:** combined pulmonary fibrosis and emphysema, deterioration, exacerbation, GAP score, lung cancer, predictors

## Abstract

Combined pulmonary fibrosis and emphysema (CPFE) patients visit hospitals frequently due to acute exacerbations (AEs); however, the predictors of CPFE AE have not been comprehensively described in literature. Thus, we investigated the predicting factors of AE in CPFE patients.

We retrospectively reviewed medical records from the past 12 years at Korea University Guro Hospital. We selected CPFE patients by computed tomography findings. Rapid deterioration (RD) was defined as acute worsening of dyspnea requiring hospitalization and the presence of newly developed radiologic abnormalities. AE was defined as RD with newly acquired bilateral pulmonary infiltrates without evidence of pulmonary infection or other known causes. We evaluated the following variables in CPFE patients: age, sex, smoking history and amount, body mass index, past medical history, pulmonary function test, gender, age, and physiology (GAP) score, and the presence of lung cancer.

Among 227 CPFE patients, 108 had RD and 31 developed AE. The most common cause of RD was infection (n = 60, 55.6%) and 28.7% (n = 31) developed AE. Lung cancer [hazard ratio (HR), 3.274; 95% confidence interval (95% CI) 1.444–7.425; *P* < .01] and GAP score (HR, 1.434; 95% CI 1.072–1.918; *P* = .02) were significant predictors of AE. The presence of lung cancer and AE were significant predictors of mortality.

In conclusion, CPFE patients with lung cancer and high GAP scores should be carefully observed for AE.

## 1 Introduction

Combined pulmonary fibrosis and emphysema (CPFE) is a recently individualized disease characterized by a predominant upper-lobe emphysema and lower-lobe fibrosis.^[1]^ Cottin et al^[2]^ first described CPFE as a distinct disease entity, which was possible to diagnose due to improvements in computed tomography (CT) scanning. It is considered to be a distinct consequence of smoking that reflects unique individual susceptibilities with its natural history.^[3]^ Thus, identification of prognostic factors of CPFE is important.

Overall, CPFE syndrome has a poor prognosis with a 5-year survival of 35% to 80%.^[4]^ Some studies reported that CPFE patients exhibited worse survival compared with patients with isolated idiopathic pulmonary fibrosis (IPF).^[5,6]^ Patients with CPFE have poor outcomes, as they are at an increased risk of acute exacerbations (AEs), sudden acceleration of disease process or superimposed acute injury, and other rapid deteriorations (RDs) caused by other conditions, including infection and heart failure, which frequently lead to death.^[1,2,7,8]^ However, the causes and incidence of RD and predictors of AE in CPFE were not comprehensively described.

Several studies identified the risk factors of AE in IPF patients.^[9–12]^ However, little is known about AE in patients with CPFE. One study found that baseline Krebs won den Lungen-6, which reflects the extent of interstitial lesion, was a useful predictor of AE in patients with CPFE.^[13]^ However, serum Krebs won den Lungen-6 is not routinely checked and there were no previous studies on other RD in patients with CPFE. Thus, in this study, we aimed to investigate the etiologies of RD and predictors of AE in patients with CPFE.

## 2 Methods

### 2.1 Study population and chest CT assessment

We retrospectively investigated medical records and CT scans at Korea University Guro Hospital, Seoul, Korea, between January 1, 2004, and December 30, 2016. Korea University Guro Hospital review board approved the study (2015GR0150). The CT imaging patterns were evaluated the same way as per Cottin et al.^[2]^ We diagnosed patients as CPFE when both of the emphysema and pulmonary fibrosis criteria were met. Emphysema was defined as the presence of well-demarcated areas of low attenuation delimitated by a very thin (>1 cm) or no wall, and/or multiple bullae (>1 cm) with upper zone predominance. Pulmonary fibrosis included reticular opacities with peripheral and basal predominance, honeycombing, architectural distortion, and/or traction bronchiectasis. We excluded patients without CT imaging or pulmonary function tests performed and those who had occupational interstitial lung disease.

### 2.2 Outcome and variables to measure

RD was defined as an acute (within 30 days) worsening of dyspnea requiring hospitalization and the presence of newly developed radiologic abnormalities.^[10]^ AE was defined as a sudden aggravation of dyspnea within 30 days with newly acquired bilateral pulmonary infiltrates without evidence of pulmonary infection or other known causes.^[11]^ Baseline clinical parameters were obtained within 1 month of the initial diagnosis. Demographic and clinical data collected included age, gender, smoking history, body mass index (BMI), comorbidities, and pulmonary function tests. For pulmonary function test, we checked physiological data including baseline and 1-year forced vital capacity volume in 1 second (FEV_1_), forced vital capacity (FVC), FEV_1_/FVC, total lung capacity (TLC), and diffusing capacity of carbon monoxide (DLco) according to the American Thoracic Society/European Respiratory Society recommendations. The results were expressed as percentages of the normal predicted values. A composite physiologic index (CPI) was calculated from the following formula: 91–(0.65 × DLco%)–(0.53 × FVC%) + (0.34 × FEV_1_%).^[14]^ Gender, age, and physiology (GAP) score was calculated using the methods suggested by Ley et al.^[15]^ Survival time was defined from the date of first diagnosis to death or to the last observation date. Similarly, AE/RD free time was defined from the date of first diagnosis to AE/RD or to the last observation date.

### 2.3 Statistical analysis

Data were presented as a mean ± standard deviations for continuous variables or percentages for categorical variables. Chi-squared and Fisher exact tests were used for categorical data, while the unpaired *t* test and Mann–Whitney *U* test were used for continuous data. Univariate Cox proportional hazard models were used to examine the association of selected variables with AE, RD, and survival. The multivariate Cox proportional hazard model using the backward elimination method was used among variables that were significant (*P* < .1) in the univariate model. Incidence of AE, RD, and survival was evaluated using a Kaplan–Meier survival curve and a log rank test. *P* values < .05 were considered statistically significant. All statistics were performed using SPSS v. 20.0 software (IBM, Chicago, IL). Our institutional review board approved the study (2015GR0150).

## 3 Results

### 3.1 Patient baseline characteristics

A total of 5118 emphysema and 1134 pulmonary fibrosis patients were screened. Among them, we identified 458 patients with both emphysema and pulmonary fibrosis. After final review based on chest CT findings, 227 CPFE patients were analyzed. The mean age was 69.4 years, mean BMI was 23.1, and 96.0% of the patients were men. All patients had a history of smoking (mean: 43.2 pack-years). During the observational period, 108 (47.6%) patients had RD, 31 (13.7%) developed AE, 61 (26.9%) were diagnosed with lung cancer, and 60 (26.4%) patients died. Baseline characteristics of patients with AE and non-RD patients are described in Table 1. There were no significant differences in the smoking amount, age, gender, and BMI between AE and non-RD group. Lung cancer was diagnosed in 38 (49.4%) patients with AE and 13 (10.9%) patients without RD (*P* < .01).

**Table 1:**
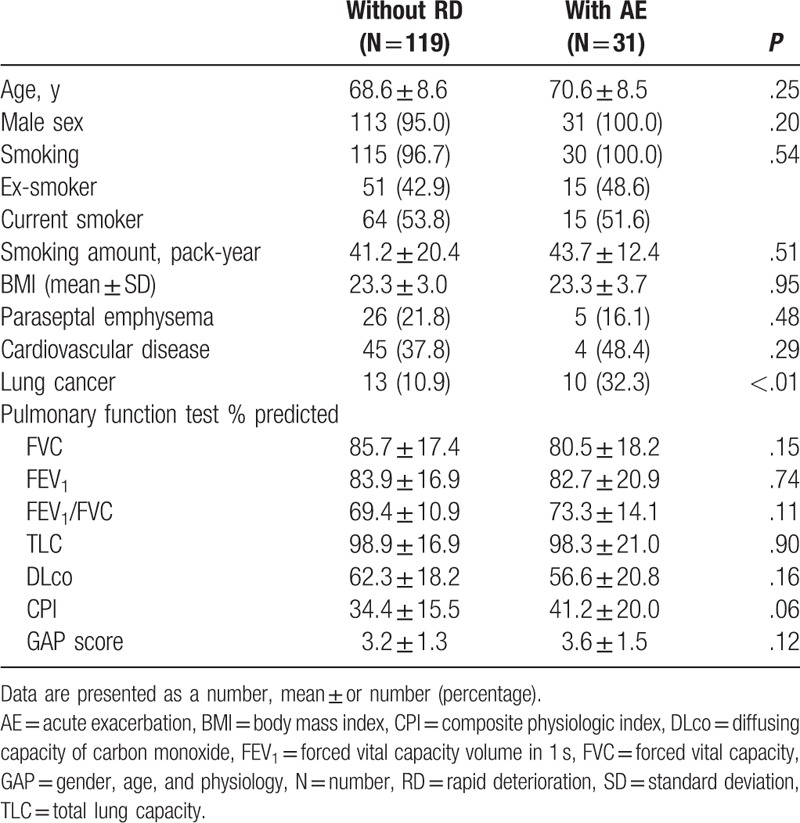
Demographic and clinical characteristics of patients without rapid deterioration and with acute exacerbation.

### 3.2 Baseline pulmonary function tests

The mean baseline FEV_1_ was 83.0%, FVC was 85.2% and FEV_1_/FVC was 69.0%. Mean DLco was 59.3%, mean CPI was 37.2, and mean GAP score was 3.4. Baseline pulmonary function tests of patients with AE and non-RD are described in Table 1. Rapid progression defined as ≥10% decrease in the percentage of predicted FVC or ≥15% decrease in the percentage of predicted DLco within 1 year was significantly more frequent in patients with AE than in patients without RD (AE 77.3% vs without RD 43.3%; *P* < .01).

### 3.3 Etiologies of RD

Median RD free time was 36.0 [95% confidence interval (95% CI) 24.9–47.1] months. The most common cause of RD was infection (60, 55.6%) and 31 (28.7%) patients developed AE. Among all patients with RD (n = 108), 91 (84.3%) had bilateral lesions and 17 (15.7%) had focal lesions. Among patients with bilateral lesion RD (n = 91), 31 (34.1%) developed AE, 46 (50.5%) developed infections (32 bacterial, 5 viral, 1 parasitic, 1 mycobacterial, and 7 unknown organism), 11 (12.1%) had heart failure, and 3 (3.3%) had pulmonary embolism. Among patients with focal lesion RD (n = 17), 14 (82.4%) developed infections and 3 (17.6%) had pneumothorax.

### 3.4 Predictors of RD

Among variables that were significant (*P* < .1) in the univariate analysis (age, smoking amount, lung cancer, FVC, and GAP score), lung cancer [hazard ratio (HR), 2.347; 95% CI 1.554–3.546, *P* < .01] and GAP score (HR, 1.291; 95% CI 1.093–1.525, *P* < .01) were significant predictors of RD in the multivariate analysis. Estimated median RD free time was 51.0 (95% CI 33.2–68.8) months in patients without lung cancer and 9.0 (95% CI 5.5–12.5) months in patients with lung cancer.

### 3.5 Incidence of AE

**Figure 1:**
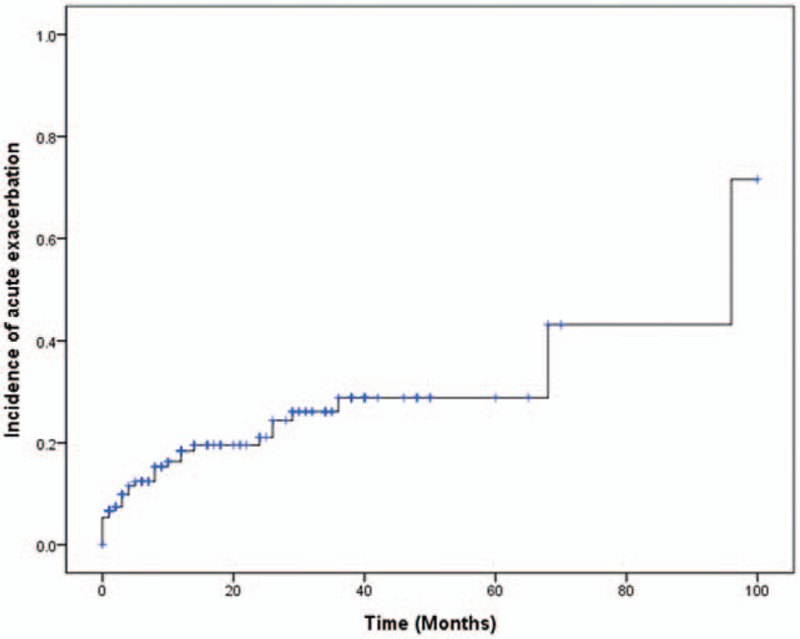
Cumulative incidence of acute exacerbation.

**Figure 2:**
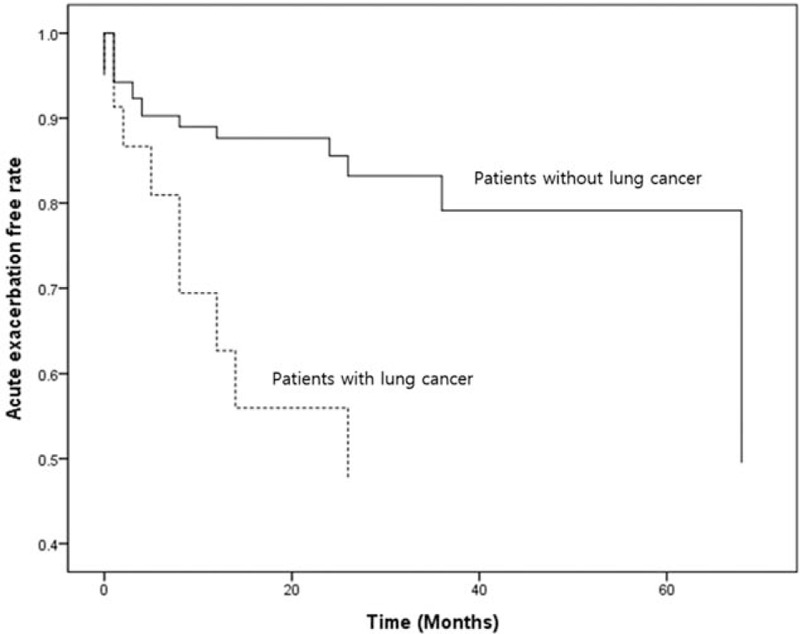
Acute exacerbation free rate curve according to the presence of lung cancer after adjusting for gender, age, and physiology score.

Median AE free time was 96.0 (95% CI 55.1–136.9) months. During the follow-up period, 31 (13.7%) patients developed AE. The Kaplan–Meier curve of the cumulative incidence of AE is demonstrated in Fig. 1. Estimated median AE free time was 96.0 (95% CI 55.1–136.9) months in patients without lung cancer and 26.0 (95% CI 2.15–49.85) months in patients with lung cancer. The AE free rate curve (adjusted for GAP score) according to the presence of lung cancer is presented in Fig. 2.

### 3.6 Predictors of AE

We performed a multivariate analysis to determine predictors of AE. Among variables that were significant (*P* < .1) in the univariate analysis (age, presence of lung cancer, FVC, and GAP score), lung cancer (HR, 3.274; 95% CI 1.444–7.425, *P* < .01) and GAP score (HR, 1.434; 95% CI 1.072–1.918, *P* = .02) were significant predictors of AE in the multivariate analysis (Table 2).

**Table 2:**
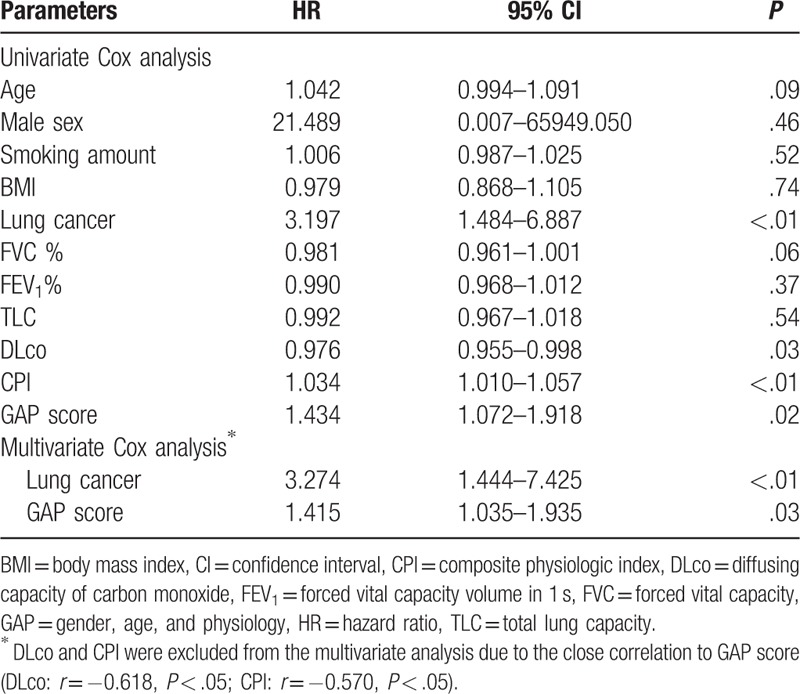
Risk factors for acute exacerbation compared with no episodes of rapid deterioration.

### 3.7 Impact of AE on overall survival

Median survival was 70.0 (33.6–106.4) months. A total of 60 (26.4%) patients died during observation period. Among the factors that were significant (*P* < .1) in the univariate analysis (presence of lung cancer, FVC, GAP score, and presence of RD), lung cancer (HR, 4.738; 95% CI 2.548–8.813; *P* < .01) and presence of AE (HR, 15.257; 95% CI 3.437–67.719; *P* < .01) or non-AE RD (HR, 13.097; 95% CI 32.434–44.529; *P* < .01) compared with non RD were significant predictors of mortality. Estimated median survival was 40.0 (95% CI 26.6–53.41) months in patients with AE and 41.0 (95% CI 23.1–58.9) months in patients with non-AE RD and 96.1 (95% CI 91.8–100.4) months in patients without RD.

### 3.8 AE in CPFE patients with lung cancer

Among 61 CPFE patients with lung cancer, 10 patients developed AE. Of those, 3 patients in early stage (1–2) lung cancer developed AE postoperatively, 5 in advanced stage (3–4) developed AE at least 4 weeks following chemotherapy, 1 patient was diagnosed with AE during the follow-up, and 1 patient developed it following pericardiocentesis for malignant pericardial effusion at diagnosis.

When we analyzed the patients with CPFE and lung cancer using multivariate cox proportional hazard models with backward elimination analysis for mortality, the presence of AE was the significant mortality predictor (AE vs non-RD: HR, 9.730; 95% CI 1.213–78.034; *P* = .03; non-AE RD vs non-RD: HR, 6.402; 95% CI 0.865–47.376; *P* = .07) rather than age, gender, FVC, GAP stage, lung cancer stage, or treatment modality.

## 4 Discussion

In our study, respiratory infection was the most common cause of RD in patients with CPFE. In addition, we found that lung cancer and GAP score were significant predictors of AE and RD, while the presence of lung cancer and AE were significant predictors of mortality.

Several studies reported a relationship between lung cancer and CPFE. Kwak et al^[16]^ showed that patients with CPFE had a higher risk of lung cancer (adjusted HR, 4.62; 95% CI 1.58–13.55) than those with emphysema alone. Similarly, Kitaguchi et al^[17]^ reported that patients with CPFE had a high risk of developing lung cancer (46%). Moreover, the presence of CPFE was a factor affecting the prognosis of lung cancer patients. Usui et al^[18]^ reported that the median overall survival of lung cancer patients who had CPFE was significantly less than that of lung cancer patients with emphysema alone. Similarly, in our study, the prevalence of lung cancer in patients with CPFE was up to 26.9%, with lung cancer identified as a prognostic factor of AE, RD, and mortality. Our results showed that the presence of lung cancer increased the probability of AE 2.7 times, RD 6.1 times, and mortality 5.7 times. Both patients with advanced and early-stage lung cancer experienced AE and RD, which led to poor outcomes. This suggests that the presence of lung cancer with its associated procedures rather than the stage was a risk factor for AE, the most significant mortality predictor in CPFE lung cancer patients. In agreement with our results, it was reported that several patients with lung cancer and CPFE presented with AE or pneumonia.^[19]^ Acute lung injury was more frequent in patients with CPFE than those with fibrosis or emphysema alone in lung cancer patients.^[18]^ Other studies reported that CPFE lung cancer patients still had poor prognosis following complete resection in earlier stage patients and those with good pulmonary functions.^[20–22]^ Although patients with CPFE often have severe dyspnea and a poor cardiopulmonary reserve resulting in low tolerance to invasive procedures^3^, those with lung cancer are often exposed to invasive diagnosis and treatment procedures. Thus, diagnostic and treatment plans should be carefully considered by assessing risk factors of AE.

In addition to presence of lung cancer, we found that GAP score was a significant independent predictor of AE in CPFE patients. In previous IPF studies, FVC and FVC changes were risk factors of AE.^[9,10,23,24]^ However, CPFE has a mixed pathophysiology of restrictive and obstructive lung defects, which can lead to near-normal values of FEV_1_ and FVC.^[2,8,25]^ Therefore, single parameters of lung function such as FVC or FEV_1_ do not reflect the severity of disease and should not be predictors of AE in CPFE. Recently, a GAP score that was based on gender, age, and 2 lung physiology tests (%FVC and % DL_CO_) was reported as a clinical prognostic factor associated with outcomes in patients with IPF.^[15]^ Several studies described that GAP score can be a predictor of AE in patients with IPF, especially in those with lung cancer.^[12,26]^ In this study, we found that GAP score was a significant predictor of AE in CPFE patients. Risk of AE in CPFE lung cancer patients can be calculated using the GAP score, which can be considered in the treatment decision making and to estimate prognosis in lung cancer patients with CPFE.

Presence of AE is a well-known risk factor of mortality in both chronic obstructive pulmonary disease (COPD) and IPF patients.^[10,27]^ We determined that AE was an important risk factor of mortality in CPFE patients. In addition, our study was the first to identify the etiology and predictors of RD in CPFE patients. A study on IPF patients reported that AE was the most frequent cause of RD followed by infection.^[10]^ In contrast, our results showed that respiratory infections were the most prevalent cause of RD in CPEE patients. This was similar to reports on COPD including pulmonary emphysema, where infections were the predominant cause of exacerbations due to several impairments in lung defenses.^[28,29]^ This may be due to CPFE being a combination of both fibrosis and emphysema, which can make CPFE patients more vulnerable to respiratory infections than IPF patients. However, predictors of AE and RD were similar (presence of lung cancer and GAP score).

Our study had some limitations. First, it was a single-center, retrospective, cohort study. Thus, prospective validation is needed. Second, we were unable to evaluate the extent of emphysema and fibrosis, which contributes to the disease progression of CPFE patients. However, this study is meaningful, as it is the first to examine the causes of RD and risk factors and impact of AE in CPFE patients.

## 5 Conclusion

Pulmonary infections were the most common causes of RD in CPFE patients followed by AE. In addition, the presence of lung cancer and a high GAP score were significant predictors of AE in CPFE patients, which affected mortality. Therefore, CPFE patients with lung cancer and a high GAP score should be carefully observed for AE.

## Acknowledgment

We thank SY Hwang for the excellent statistical support.

## Author contributions

**Conceptualization:** Jee Youn Oh, Young Seok Lee, Kyung Hoon Min, Gyu Young Hur, Sung Yong Lee, Kyung Ho Kang, Jae Jeong Shim.

**Data curation:** Jee Youn Oh, Young Seok Lee, Kyung Hoon Min, Gyu Young Hur, Sung Yong Lee, Kyung Ho Kang, Jae Jeong Shim.

**Formal analysis:** Jee Youn Oh, Young Seok Lee, Kyung Hoon Min, Gyu Young Hur, Sung Yong Lee, Kyung Ho Kang, Jae Jeong Shim.

**Investigation:** Jee Youn Oh, Young Seok Lee, Kyung Hoon Min, Gyu Young Hur, Sung Yong Lee, Kyung Ho Kang, Jae Jeong Shim.

**Methodology:** Jee Youn Oh, Young Seok Lee, Kyung Hoon Min, Gyu Young Hur, Sung Yong Lee, Kyung Ho Kang, Jae Jeong Shim.

**Project administration:** Jee Youn Oh, Young Seok Lee, Kyung Hoon Min, Gyu Young Hur, Sung Yong Lee, Kyung Ho Kang, Jae Jeong Shim.

**Resources:** Jee Youn Oh, Young Seok Lee, Kyung Hoon Min, Gyu Young Hur, Sung Yong Lee, Kyung Ho Kang, Jae Jeong Shim.

**Software:** Jee Youn Oh, Young Seok Lee, Kyung Hoon Min, Gyu Young Hur, Sung Yong Lee, Kyung Ho Kang, Jae Jeong Shim.

**Supervision:** Jee Youn Oh, Young Seok Lee, Kyung Hoon Min, Gyu Young Hur, Sung Yong Lee, Kyung Ho Kang, Jae Jeong Shim.

**Validation:** Jee Youn Oh, Kyung Hoon Min, Gyu Young Hur, Sung Yong Lee, Kyung Ho Kang, Jae Jeong Shim.

**Visualization:** Jee Youn Oh, Kyung Hoon Min, Gyu Young Hur, Sung Yong Lee, Kyung Ho Kang, Jae Jeong Shim.

**Writing – original draft:** Jee Youn Oh, Kyung Hoon Min.

**Writing – review & editing:** Jee Youn Oh, Sung Yong Lee, Kyung Ho Kang, Jae Jeong Shim.
